# The Effect of an EHR Order Set on Cancer Screening Order Rates in Community-Based Health Centers

**DOI:** 10.1055/a-2524-5076

**Published:** 2025-06-04

**Authors:** Rachel Gold, Constance Owens-Jasey, Jean O'Malley, Rose Gunn, April Lee, Matthew Jones, Michaella Latkovic-Taber, Gordon Barker, Nathalie Huguet

**Affiliations:** 1 Research Department, OCHIN Inc., Portland, Oregon, United States; 2 BRIDGE-C2 Implementation Science Center for Cancer Control, Oregon Health & Science University, Portland, Oregon, United States; 3 Kaiser Permanente Northwest Center for Health Research, Portland, Oregon, United States; 4 Department of Family Medicine, Oregon Health & Science University, Portland, Oregon, United States

**Keywords:** cancer prevention, electronic health record, mixed methods

## Abstract

**Objectives:**

Adoption of electronic health record (EHR)-based clinical decision support tools in community-based health centers might increase the provision of indicated cancer screening orders. We examined: (1) if the use of the care gaps smartset (CGS), an EHR tool that expedites ordering care, is associated with colorectal/cervical cancer (CRC/CVC) screening order rates; and (2) how selected implementation strategies, barriers, and facilitators impact CGS use.

**Methods:**

Within a sequential mixed methods design, we used multivariate regression to assess associations between clinic- and provider-level CGS use and cancer screening order rates. Tool use rates (3/2018–12/2023) were measured as the rate of encounters at which any orders were placed via the CGS and then categorized by use level. Surveys ( *n* = 81) and semi-structured interviews ( *n* = 11) with clinic staff assessed strategies to improve tool use.

**Results:**

Clinics and providers that ever used the CGS had higher CRC screening order rates than non-users. Higher CGS use was associated with better CRC screening order rates. By 12/2023, CRC screening orders were 4.4% ( *p* < 0.05) higher in high-use clinics versus those with no CGS use. CGS use was not associated with CVC screening order rates. Qualitative findings indicate effective CGS use was enhanced by leadership support, clear workflows, and clinic-led training. Barriers to CGS use included low user awareness of/trust in the tool, and tool functions that were not optimized.

**Conclusion:**

CGS use can support cancer screening ordering; its adoption may be enhanced by varied training approaches and workflow design.

## Background and Significance

Screening enables the early detection of cervical (CVC) and colorectal (CRC) cancers. Yet in the United States, many healthcare providers fall short of Healthy People 2030 screening targets (CVC 84%, CRC 68%), 1 2 3 4 especially those serving marginalized populations, such as community-based health centers (CHCs). 5 A recent analysis found that in 2019, only 50% of eligible CHC patients were up to date on CVC screening and 44% on CRC screening. 5 One of the key barriers to CHCs providing these screenings is that clinic staff have inadequate time at a brief visit to identify all of a patient's preventive care needs and place orders to address those needs, and CHCs have inadequate staff and resources to conduct outreach between visits to patients who are overdue for screenings. 6

Clinical decision support tools within electronic health records (EHRs) hold promise for helping care teams systematically follow cancer-related care guidelines. 7 8 9 10 11 Use of such tools can improve the provision of other evidence-based treatment elements by identifying patient “care gaps” and expediting the ordering of these care elements. 9 12 13 14 15 However, a recent review identified substantial knowledge gaps regarding the effectiveness and adoption of such tools in the context of cancer screening provision. 16 Evidence also shows that barriers to clinical decision support tool adoption 17 18 include inadequate staff knowledge about potentially useful EHR tools and how to optimize their use to expedite screening order provision. 19 Little is known about how to address these barriers; knowledge of how to improve the adoption of effective clinical decision support tools is needed.

This study describes the adoption and effectiveness of one such tool after its activation in an EHR shared by a national network of CHCs. Health maintenance, also called care gaps, is a suite of tools in the Epic EHR, including point-of-care alerts when a patient is indicated for a given care element. The tools' content reflects clinical guidelines including needed care steps related to cancer screening from the United States Preventive Services Task Force and Centers for Disease Control and Prevention and secondary sources relevant to cancer screening (e.g., American Society of Colposcopy and Cervical Pathology's cervical cancer screening abnormality tracking).

OCHIN, Inc. is a health information technology consultancy whose member CHCs (in this study period, 1,776 clinic sites) share a single instance of the Epic EHR which OCHIN regularly modifies to meet CHCs' needs. The care gaps smartset (CGS; also called an order set) was built at OCHIN to enhance Epic's health maintenance tools, activated in OCHIN's EHR in 03/2019, and made available to all users of this EHR ( Supplementary Fig. S1 , available in the online version only). The CGS improves on the EHR's standard interface for reviewing care gaps and its interface for placing orders, which are in separate locations in the EHR, by listing all care gaps and enabling one-click orders to address each gap, in one interface. This is meant to increase efficiency by reducing the steps and time needed to place orders because current workflows require placing screening orders by navigating to other EHR sections and entering individual orders one at a time.

## Objective

The CGS is a point-of-care tool, so these analyses focused on cancer screening order provision, which usually occurs at the encounter. We assessed whether clinic- and provider-level CGS use was associated with order rates of guideline-concordant cancer screening, then evaluated knowledge of this tool among potential users and identified facilitators and barriers to CGS adoption. Results can inform the development and adoption of the CGS and similar clinical decision support tools with the potential to improve cancer screening rates in CHCs and other primary care settings.

## Methods

This study's sequential mixed methods design included analyzing EHR data to characterize cancer screening orders and CGS use, followed by descriptive survey data and semi-structured interviews assessing clinic staff implementation and use of the CGS to order cancer screenings.

### Quantitative Data and Analyses

The quantitative sample comprised 180 health systems including 1,776 clinics. Data from OCHIN's Epic EHR were made research-ready by the Accelerating Data Value Across a National Community Health Center Network (ADVANCE) Clinical Research Network, a member of PCORnet, and supplemented with CGS use data.

The study period was 3/1/2018–12/31/2023 (1-year pre-CGS activation through 4 years post-activation). Monthly order rates for eligible patients in year 0 (03/2018–02/2019, pre-tool activation) were compared to those in year 1 (03/2019–02/2020), year 2 (broken into 03/2020–07/2020 and 08/2020–02/2021 to isolate varying pandemic effects), year 3 (03/2021–02/2022), and year 4 (03/2022–12/2023). Clinics were included in the study for any month in which they had ≥10 patients in the CRC or CVC screening measure denominators. Data on CRC and CVC screening, human papillomavirus (HPV) testing, and screening criteria were collected on all clinic patients meeting the age and sex criteria for related quality measures. 20 21

Use (adoption) of the CGS during the study period was evaluated at the clinic and provider levels. Its use rates were defined as the percentage of encounters at which cancer screening-eligible patients received any order through the CGS, rather than cancer-specific orders, because the CGS is designed to promote ordering multiple care elements at once. As such, CGS used to order any screening (e.g., lipid screening) could prompt users to order other due screenings. Clinic-level CGS use rates were categorized as 0 (no CGS use), 1 (low use: any use below the top quartile), and 2 (high use: top quartile of use levels). Provider-level CGS use rates were categorized as 0 (no CGS use), 1 (low use: usage below median), 2 (moderate use: above median but below the top quartile), and 3 (high use: top quartile of use levels). Usage quartiles were set by CGS usage level in the study's final year. In the clinic-level analysis, the second and third quartiles were combined due to low clinic numbers in the third quartile.

Tool effectiveness was assessed as clinic- and provider-level CRC and CVC screening order rates measured as each month's percent of patients due for CRC or CVC screening at an encounter who received an indicated order within a month of that encounter. Patients due for screening were those who were not up to date on a given encounter; a patient was considered up to date only if they had an order with “completed” status or a satisfied health maintenance entry indicating that the screening occurred. CRC screening orders included fecal immunochemical tests (FIT), fecal occult blood tests (FOBT), flexible sigmoidoscopy, colonoscopy, and computed tomography colonography. Federal criteria for up-to-date CVC status for women over 30 changed during the study period, so up-to-date status was determined by the date of and patient age at the encounter. CVC screening orders were captured for patients due for Pap tests (<30 years old and ≥30 years old) and for HPV tests (≥30 years old), per guidelines.

Clinic- and provider-level screening order rates' association with CGS use category were estimated using Poisson regression models with robust sandwich estimators to adjust for the correlation of rates within the clinic over time. Models were adjusted for how long a clinic had been on the OCHIN EHR to account for differential familiarity with EHR functions. Associations between monthly CGS use level and CRC and CVC order rates were summarized for each year post-CGS activation. Because CVC screening recommendations differ by age, CVC analyses were stratified by age, with Pap and HPV order rates assessed separately in women 30 years and older. Quantitative analyses used SAS Enterprise Guide Version 8.4.

### Survey Data and Analysis

An opportunistic poll survey was conducted during an OCHIN Clinical Operations Review Committee meeting in 05/2023, which included 240 attendees representing clinics across the OCHIN network; 81 (34%) attendees participated. The survey asked about respondents' experience with the CGS and featured Likert scale questions on familiarity with the CGS (not at all, somewhat, very), frequency of use (never, sometimes, as often as possible), time of use (not used, used by someone else on care team, before, during, after patient visit), barriers to use (e.g., too many clicks, lack of knowledge), and perceived usability (e.g., efficient, user friendly). Summary statistics were used.

### Qualitative Data and Analyses

We interviewed staff from five CHCs purposively selected based on having high rates of both CGS use and of CRC/CVC guideline-concordant cancer screening orders, to optimize the likelihood that interviewees had CGS use experience and could share their perspectives on the tool's utility in practice, reflecting on challenges and adaptations over time. We interviewed 11 clinic staff representing roles of those most involved in reviewing due/overdue care gaps and placing appropriate orders from 10/2023 through 01/2024. Interviewees included two-panel managers, four nurse practitioners, two medical doctors, one lead medical assistant, one physician assistant, and one director of care quality.

Informed by implementation science and human-centered design principles, 22 interviews included questions about strategies clinics used to support CGS adoption and a “guided tour” in which interviewees demonstrated how they typically use the CGS or other order sets to address care gaps. The interviews probed on the perceived strengths of these tools, areas for improvement, and strategies employed to encourage their use. Interviews were recorded and transcribed professionally for analysis.

**Fig. 1:**
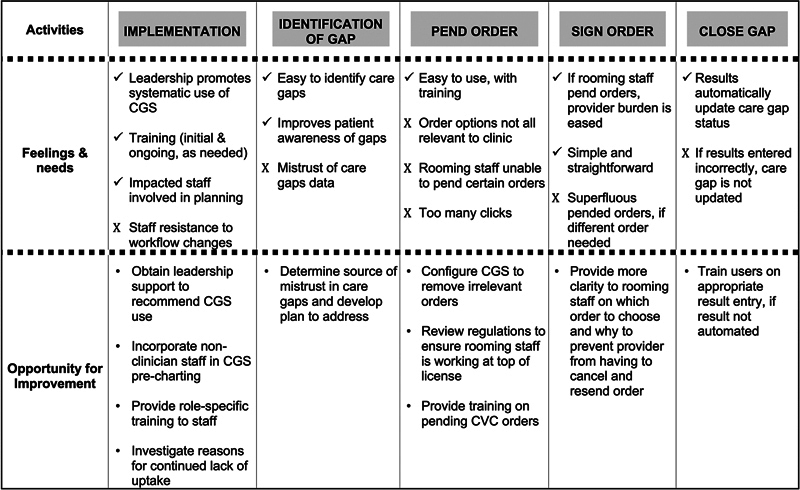
Summary of care gap smartset use barriers (X) and facilitators (✓) and opportunities for improvement.

Transcripts were analyzed using a rapid analytic approach 23 24 guided by the Integrated Technology Implementation Model (ITIM), 25 which combines implementation science and health information technology domains. First, interview transcripts were summarized using a template organized by domains corresponding to study research questions and the ITIM. Completed summary domains were checked by a second qualitative team member; inconsistencies were resolved. Next, summary data from each domain were transferred to a matrix table for analyzing data across interviews, including identifying potential themes and key points. Early impressions were brought to the multidisciplinary study team to support interpretation and prioritize themes. The analytic approach culminated in the development of a journey map ( Fig. 1 ) that incorporated different perspectives of CGS users, scenarios in which it is utilized, the implementation and tool adoption journey, and behaviors and users' thoughts on CGS implementation and use. The resulting journey map and findings narrative were reviewed by interview participants to confirm interpretation and strengthen validity.

## Results

Table 1 displays the characteristics of the sample and the number and percent of clinics and providers by CGS use category for each study period. Overall 57% of the patients served in these clinics were female, more than 60% represented ethnic and racial minority groups, and nearly 50% were aged 30–64 years. Patient demographic characteristics do not change over time. Table 1 also shows the increasing number of clinics and providers over time in the network.

**Table 1:** Characteristics of study sample and CGS smartset use over time

	Pre-CGS	Post-CGS activation				
	03/2018–02/2019	03/2019–02/2020	03/2020–07/2020	08/2020–02/2021	03/2021–02/2022	03/2022–12/2023
Clinics in CRC sample, *n*	458	608	620	655	818	939
No use, %	100	78.3	81.9	79.2	71.8	73.8
Low use,%	0	16.1	12.3	10.4	14.4	13.7
High use, %	0	5.6	5.8	10.4	13.8	12.5
Clinics in CVC sample (age <30 y), *n*	427	553	584	601	760	890
No use, %	100	76.1	81.9	79.2	71.8	73.8
Low use,%	0	17.4	12.3	10.4	14.4	13.7
High use, %	0	6.5	3.2	4.7	6.2	6.0
Clinics in CVC sample (age ≥30 y), *n*	465	614	619	655	818	967
No use, %	100	78.5	81.9	79.2	71.9	74.7
Low use,%	0	16.0	12.3	10.4	14.3	13.3
High use, %	0	5.5	5.8	10.4	13.8	12.0
Providers in CRC sample, *n*	9,849	12,412	11,846	12,564	17,910	20,107
No use, %	100	97.8	97.6	95.8	94.8	95.2
Low use, %	0	1.6	1.7	2.3	2.8	2.4
Moderate use, %	0	0.3	0.4	0.7	0.9	1.0
High use, %	0	0.3	0.3	1.1	1.5	1.4
Providers in CVC sample (<30 y), *n*	1,578	1,886	2,009	2,058	3,028	3,082
No use, %	100	96.02	96.02	91.35	89.3	89.68
Low use, %	0	3.39	3.38	5.39	6.14	5.48
Moderate use, %	0	0.16	0.25	1.6	1.85	2.37
High use, %	0	0.42	0.35	1.65	2.71	2.47
Providers in CVC sample, *n* (≥30 y)	4,680	5,801	5,752	5,912	8,523	8,725
No use, %	100	96.38	96.44	93.54	91.69	92.09
Low use, %	0	2.95	2.89	4.09	5.01	4.55
Moderate use, %	0	0.38	0.38	1	1.3	1.44
High use, %	0	0.29	0.3	1.37	1.99	1.91
Patients characteristics						
Female, a %	57	57	57	57	57	57
Black, non-Hispanic, %	18	18	17	17	16	16
White, non-Hispanic, %	39	34	34	34	33	32
Other race, non-Hispanic, %	6	8	8	8	8	8
Hispanic, %	32	34	34	34	36	37
Unknown race or ethnicity, %	5	5	6	7	7	8
Age <21, %	28	30	26	25	28	28
Age 21 to <30, %	13	12	13	12	13	13
Age 30–64, %	49	47	49	49	47	47
Age ≥65, %	10	11	13	13	12	12

Abbreviations: CGS, care gaps smartset; CRC, colorectal; CVC, cervical.

a Excludes patients of unknown sex. The year 2020 was broken into 3/2020–7/2020 and 8/2020–2/2021 to isolate varying pandemic effects. Clinic-level CGS use rates were categorized as 0 (no CGS use), 1 (low use: any use below the top quartile), and 2 (high use: top quartile of use levels). Provider-level CGS use rates were categorized as 0 (no CGS use), 1 (low use: usage below median), 2 (moderate use: above median but below the top quartile), and 3 (high use: top quartile of use levels).

### Quantitative Results—CRC Orders

**Fig. 2:**
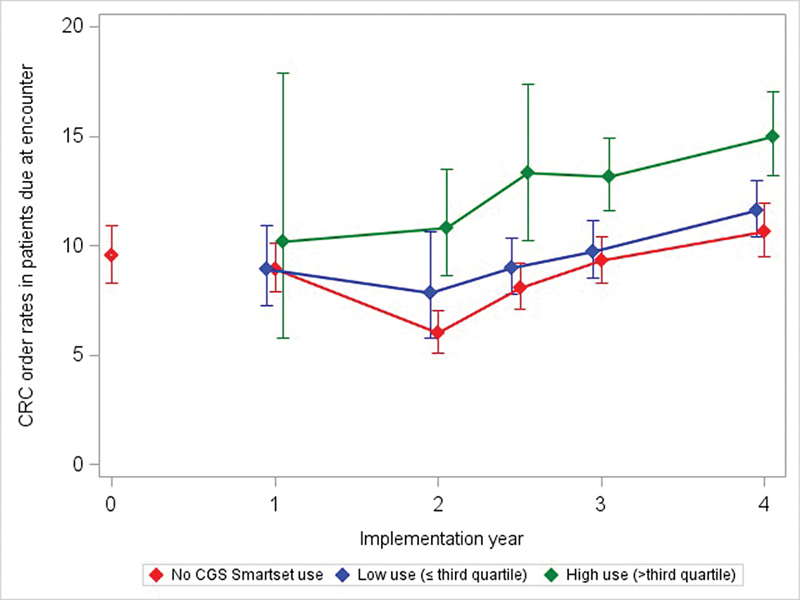
Clinic-level CGS use and colorectal cancer screening order rates, 2018–2023. Implementation year is defined by the year start date as follows; 0 = 03/2018–02/2019 with 458 clinics (before the CGS tool became available), 1 = 03/2019–02/2020 with 608 clinics, 2 = 03/2020–07/2020 with 655 clinics (year 2.5 = 08/2020–02/2021, 655 clinics), 3 = 03/2021–02/2022 with 818 clinics, and 4 = 03/2022–12/2023 with 939 clinics. Mean CRC order rates for the Implementation year (% patients with orders placed within 30 days of an encounter when due for CRC screening) were modeled from a Poisson regression of monthly rates adjusting for clinics in their first year on EHR and accounting for within clinic correlation using robust sandwich estimators. CGS, care gaps smartest; CRC, colorectal; EHR, electronic health record.

CRC screening order rates prior to CGS tool activation (03/2019) were <10% of patients due at encounters ( Fig. 2 , implementation year 0). The rate declined in 2020, then increased in 2021 and 2022. Clinic-level CGS use for placing any orders was associated with a higher CRC screening order provision rate. Clinics with the highest CGS use had significantly higher CRC screening order rates compared to no or low CGS use in years 2–4 post-implementation ( Table 2 ). By the end of the study period, 03/2022–12/2023, screening order rates in clinics with the highest CGS use (CRC rate = 15%) was 3.4 percentage points (relative rate = 1.29, 95% confidence interval [CI] = 1.15–1.44) higher than in clinics with lower use (CRC rate = 12%) and 4.4 percentage points (relative rate = 1.41, 95% CI = 1.25–1.59) higher than in clinics with no CGS use (CRC rate = 11%).

**Table 2:** Relative rate of using CGS to order cancer screenings over time

	03/2018–02/2019	03/2019–02/2020	03/2020–07/2020	08/2020–02/2021	03/2021–02/2022
Clinic-level CRC					
Low vs. none	1.00 (0.86–1.16)	1.31 (0.99–1.72)	1.11 (0.99–1.25)	1.05 (0.95–1.16)	1.09 (1.01–1.18)
High vs. none	1.14 (0.64–2.00)	1.80 (1.42–2.28)	1.65 (1.28–2.13)	1.42 (1.26–1.59)	1.41 (1.25–1.59)
High vs. low	1.14 (0.65–2.01)	1.38 (1.02–1.85)	1.49 (1.11–1.98)	1.35 (1.19–1.53)	1.29 (1.15–1.44)
Clinic-level CVC <30 y					
Low vs. none	0.97 (0.61–1.55)	1.05 (0.70–1.56)	0.99 (0.61–1.59)	1.10 (0.81–1.51)	0.85 (0.55–1.32)
High vs. none	0.60 (0.26–1.41)	0.55 (0.08–3.56)	1.35 (0.49–3.67)	0.61 (0.14–2.56)	0.84 (0.23–3.15)
High vs. low	0.62 (0.21–1.83)	0.52 (0.08–3.37)	1.36 (0.60–3.12)	0.55 (0.14–2.17)	0.99 (0.28–3.47)
Clinic-level CVC ≥30 y					
Low vs. none	0.95 (0.73–1.23)	1.15 (0.89–1.48)	0.86 (0.62–1.20)	0.97 (0.78–1.20)	0.98 (0.74–1.29)
High vs. none	0.86 (0.50–1.49)	0.95 (0.38–2.39)	0.95 (0.41–2.18)	0.59 (0.24–1.45)	0.89 (0.38–2.10)
High vs. low	0.90 (0.46–1.77)	0.83 (0.33–2.09)	1.10 (0.57–2.12)	0.61 (0.28–1.33)	0.92 (0.43–1.95)
Provider level CRC					
Low vs. none	1.11 (0.99–1.25)	1.40 (1.21–1.63)	1.23 (1.10–1.37)	1.14 (1.07–1.22)	1.18 (1.09–1.27)
Moderate vs. none	1.61 (1.34–1.94)	2.51 (1.98–3.17)	2.07 (1.86–2.30)	1.78 (1.65–1.93)	1.59 (1.48–1.71)
High vs. none	1.45 (1.21–1.74)	1.79 (1.38–2.31)	1.69 (1.49–1.91)	1.56 (1.42–1.72)	1.35 (1.24–1.47)
Moderate vs. low	2.51 (1.98–3.17)	3.14 (2.38–4.16)	3.01 (2.61–3.48)	2.45 (2.23–2.70)	2.08 (1.91–2.27)
High vs. low	2.25 (1.75–2.90)	2.24 (1.66–3.03)	2.45 (2.08–2.89)	2.15 (1.92–2.41)	1.77 (1.60–1.95)
High vs. moderate	1.56 (1.22–1.98)	1.25 (0.97–1.62)	1.45 (1.27–1.66)	1.38 (1.27–1.50)	1.31 (1.22–1.41)
Provider-level CVC <30 y					
Low vs. none	1.15 (0.82–1.62)	1.51 (1.02–2.22)	1.31 (0.99–1.73)	0.79 (0.59–1.05)	0.68 (0.41–1.14)
Moderate vs. none	1.64 (0.55–4.84)	2.86 (1.26–6.48)	1.15 (0.49–2.72)	1.19 (0.66–2.13)	0.74 (0.42–1.29)
High vs. none	1.42 (0.54–3.75)	1.90 (0.91–3.95)	0.88 (0.37–2.10)	1.50 (0.83–2.73)	1.08 (0.56–2.09)
Moderate vs. low	0.93 (0.42–2.02)	1.37 (0.62–3.03)	2.70 (1.35–5.42)	0.90 (0.54–1.48)	1.30 (0.78–2.18)
High vs. low	0.80 (0.35–1.86)	0.91 (0.40–2.08)	2.06 (1.01–4.22)	1.14 (0.66–1.95)	1.91 (0.97–3.75)
High vs. moderate	0.57 (0.14–2.25)	0.48 (0.20–1.18)	2.35 (0.89–6.23)	0.76 (0.41–1.40)	1.76 (0.89–3.49)
Provider-level CVC ≥30 y					
Low vs. none	0.87 (0.57–1.32)	1.45 (0.99–2.10)	1.13 (0.86–1.48)	0.87 (0.70–1.09)	0.77 (0.63–0.96)
Moderate vs. none	1.55 (0.80–2.98)	0.44 (0.11–1.70)	1.39 (0.81–2.40)	0.91 (0.63–1.31)	0.83 (0.57–1.21)
High vs. none	1.77 (0.84–3.74)	0.31 (0.08–1.20)	1.23 (0.69–2.19)	1.04 (0.71–1.52)	1.07 (0.74–1.55)
Moderate vs. low	0.77 (0.28–2.13)	1.12 (0.20–6.21)	2.05 (1.21–3.46)	0.99 (0.70–1.42)	1.04 (0.69–1.56)
High vs. low	0.88 (0.29–2.67)	0.78 (0.16–3.82)	1.81 (1.02–3.22)	1.14 (0.78–1.66)	1.34 (0.86–2.08)
High vs. moderate	0.50 (0.14–1.72)	2.53 (0.45–14.41)	1.47 (0.82–2.63)	1.09 (0.74–1.61)	1.25 (0.77–2.04)

Abbreviations: CGS, care gaps smartset; CRC, colorectal; CVC, cervical.

Note: The year 2020 was broken into 3/2020–7/2020 and 8/2020–2/2021 to isolate varying pandemic effects. Clinic-level CGS use rates were categorized as 0 (no CGS use), 1 (low use: any use below the top quartile), and 2 (high use: top quartile of use levels). Provider-level CGS use rates were categorized as 0 (no CGS use), 1 (low use: usage below median), 2 (moderate use: above median but below the top quartile), and 3 (high use: top quartile of use levels).

Providers with low, moderate, and high CGS use had higher CRC screening order rates than those with no use in years 2–4 post-implementation ( Table 2 ; Supplementary Fig. S2 , available in the online version only). By the end of the study period, 03/2022–12/2023, among providers with the highest CGS use (CRC rate = 23%), the screening order rate was 5.6 percentage points (relative rate = 1.31, 95% CI = 1.22–1.41) higher than among providers with moderate use (CRC rate = 18%), 10.1 percentage points (relative rate = 1.77, 95% CI = 1.60–1.97) higher than providers with low use (CRC rate = 13%), and 12.1 percentage points (relative rate = 1.35, 95% CI = 1.24–1.47) higher than providers with no CGS use (CRC rate = 11%). Among providers with moderate CGS use (CRC rate = 18%), the screening order rate was 4.6 percentage points (relative rate = 2.8, 95% CI = 1.91–2.27) higher than in providers with low use (CRC rate = 13%) and 6.7 percentage points (relative rate = 1.59, 95% CI = 1.48–1.71) higher than in providers with no use (CRC rate = 11%). Lastly, among providers with low CGS use (CRC rate = 13%), the screening order rate was 2 percentage points (relative rate = 1.18, 95% CI = 1.09–1.27) higher than in providers with no use (CRC rate = 11%).

### Quantitative Results—CVC Orders

**Fig. 3:**
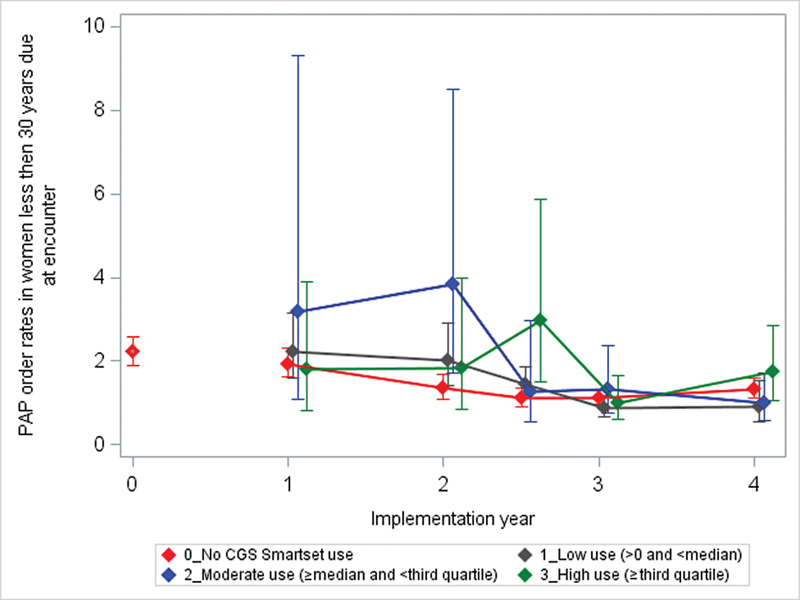
Clinic-level CGS use and cervical cancer screening order rates for women (age <30 years), 2018–2023. Implementation year is defined by the year start date as follows; 0 = 03/2018–02/2019 with 427 clinics (before the CGS tool became available), 1 = 03/2019–02/2020 with 553 clinics, 2 = 03/2020–07/2020 with 584 clinics (year 2.5 = 08/2020–02/2021, 601 clinics), 3 = 03/2021–02/2022 with 760 clinics, and 4 = 03/2022–12/2023 with 890 clinics. Mean CVC order rates for the implementation year (% of patients with order placed within 30 days of an encounter when due for cervical cancer screening) were modeled from Poisson regression of monthly rates adjusting for providers in their first year on EHR and accounting for within provider correlation using robust sandwich estimators. CGS, care gaps smartest; CVC, cervical cancer; EHR, electronic health record.

**Fig. 4:**
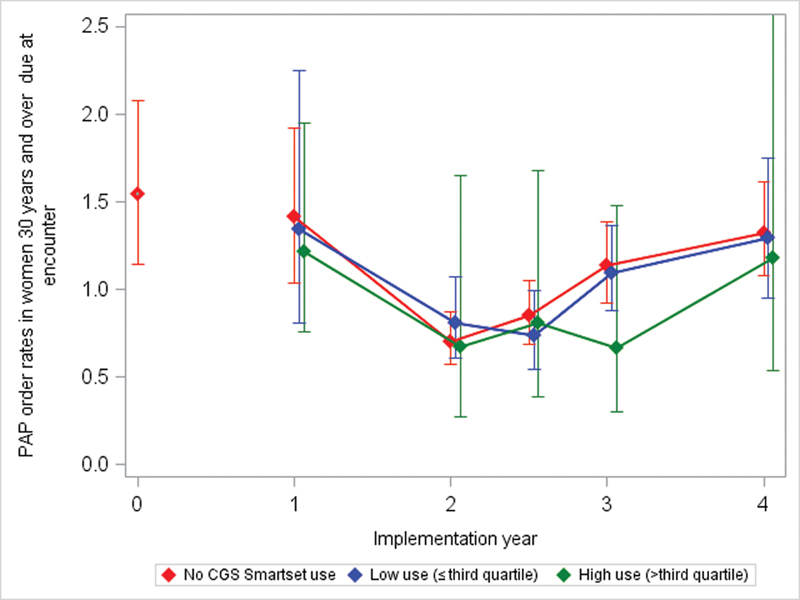
Clinic-level CGS use and cervical cancer order rates for women (age ≥30 years), 2018–2023. Implementation year is defined by year start date as follows; 0 = 03/2018 with 465 clinics (before the CGS tool became available), 1 = 03/2019 with 614 clinics, 2 = 03/2020 with 655 clinics, 3 = 03/2021 with 818 clinics, and 4 = 03/2022 with 967 clinics. Mean PAP order rates for the implementation year (% of patients with order placed within 30 days of an encounter when due for cervical cancer screening) were modeled from Poisson regression of monthly rates adjusting for providers in their first year on EHR and accounting for within provider correlation using robust sandwich estimators. CGS, care gaps smartest; EHR, electronic health record.

As seen in Figs. 3 and 4 , Table 2 , and Supplementary Figs. S3 and S4 (available in the online version only), no association was seen between CGS use and CVC screening orders.

### Survey Results

The majority of survey respondents (59%) were somewhat/very familiar with the CGS, and 56% reported that their care team used the CGS sometimes/as often as possible; 51% of users reported that someone else on the care team used it and 65% reported using it during the visit ( Table 3 ). Users reported that medical assistants often use the CGS to pend orders when starting a patient visit, and others reported its usefulness for support staff. The most common reasons for CGS use were efficiency, allowing support staff to pend orders as motivating tool use, and reported ease of use as doing so. The most common barriers to use included the tool's display being cluttered and that the tool was not well-configured to clinic needs. Over a third reported not knowing that the tool existed.

**Table 3:** Summary of survey responses of OCHIN member health centers' perceptions of CGS

	*n*	%
Familiarity with CGS ( *n* = 81)		
Not at all familiar	33	41
Somewhat or very familiar	48	59
Does the provider's clinic use CGS ( *n* = 62)		
No	27	44
Yes	35	56
Who in the clinic uses CGS ( *n* = 37)		
Someone else in the care team	19	51
Responding provider	17	49
When does the provider use CGS ( *n* = 17)		
Before the visit	5	29
During the visit	11	65
After the visit	2	12
Most common reasons for using CGS a ( *n* = 34)		
It is efficient	14	41
It allows support staff to pend orders	14	41
It is user friendly/easy-to-use interface format	11	32
Most common reasons for not using CGS a ( *n* = 53)		
Did not know about it	21	40
Too cluttered; it presents too many options for orders	14	26
Not configured to my needs	12	23
Too many clicks	5	9
Functions do not meet my needs	5	9

Abbreviation: CGS, care gaps smartset.

a Respondents could provide multiple responses, percentages do not total 100%.

### Interview Results

Themes emerged from the interviews regarding training and leadership support, workflow design and associated roles, and characteristics of the technology ( Fig. 1 ).

#### Leadership Support and Training

Participants reported starting using the CGS either because they received training during their clinic's EHR initial rollout or by “discovering” it in the EHR. Three clinics' leadership formally established an expectation of CGS use among relevant staff and developed workflows and training involving support staff (e.g., medical assistants and panel managers) working closely with providers. These initiatives often involved role-specific training, an EHR demonstration, hands-on practice, and ad-hoc refresher training. One physician's assistant described their CGS training approach,

*We found that it helps to have somebody in the training team that we have with each department because while our informatics team has a lot of just general Epic knowledge, just to know the nitty-gritty of how our organization does things has helped. So we've recruited somebody from behavioral health and somebody from women's health to give their expertise on each thing. And I think, then, that person going back and presenting to their specific department gives a little more credit to the presenter when they're actually using it as well.* [CHC 2]

Interviewees cited leadership support, including a formalized expectation of CGS adoption and role-specific training, as driving CGS uptake. When describing the motivation of leaders for systematic CGS use, an MD shared,

*Being on some of the central leadership meetings, there was a move to see how we could shift some of our preventative care goals away from the provider, with the thought that if the provider is not having to do them in the visit with the 18 other priorities that are going on, and if they were more auto populated, that perhaps we would see better rates in our patient populations of having those screenings done in a timely fashion. And then when we launched it, it was via a grand rounds where all role groups were present to be trained in their different role within that system.* [CHC 4]

#### Workflow

Three clinics formally incorporated non-clinician staff in their CGS use workflows. Their rooming/panel management staff were responsible for CGS pre-charting (reviewing care gaps for upcoming patients, pending cancer screening orders) as allowable. Orders were then signed by the provider. These workflows differed for CVC screening: given the complexity of these order options (Pap vs. HPV testing), non-clinician staff generally did not pend CVC orders. Two clinics had workflows in which the providers were responsible for all components of the CGS orders. One nurse practitioner shared,

*We do have a litany of, “Use the Care Gap SmartSet. Use the Care Gap SmartSet.” And then I think people realize that it's helpful. It is a collection of all the orders that you need. And you can do it in pre-charting and then pend your orders. You can look at it to get an idea of what needs to be ordered in the room. I think that we just really have hit people over the head with it with, “Why wouldn't you want to use this?” And it seems to be working fairly well.* [CHC 2]

#### Usability

Participants identified usability and accessibility as impacting CGS use. The CGS expedites ordering by providing a centralized place to order all needed preventive health elements, set up to require minimal medical decision-making, and support team-based care. At clinics with standardized CGS use, the provider burden was mitigated by team members' pre-charting activities. A medical director described this,

*It really guides [the MAs] through such that they're not having to make a lot of medical decisions, but that all those orders that show up for me to sign, I glance at, but I don't have to do too, too much critical thinking about because it's pretty in line with what we would expect them to be. So I just really like that it takes that burden off the visit, and I can focus more on what the patient has identified that they're coming in for and still make sure that even though this might not be their top priority, that we're at least still presenting the things that are important for their preventative care and screening, which always feels like it falls off the back end when there's a lot of other things to prioritize.* [CHC 4]

Participants reported some challenges. Some felt the CGS order form requires many clicks to expand, and that order entries listed therein were not always applicable to the clinic. There were some technical challenges related to how information is presented (e.g., the inclusion of unnecessary health maintenance topics). While CGS pre-charting was mostly viewed as time saving, pended orders became burdensome if the clinician had to change them. Even when pended per protocol, order type and lab locations can change based on patient insurance type and preference, yielding additional work to replace the pended order. A medical director commented,

*And then if [the patient] shows up and they actually have a different coverage or lost their coverage or got their coverage, then you're kind of pivoting to be like, “Okay, I have to change the lab location.” And so there's only so much that you can pre-chart until the day that they actually show up.* [CHC 5]

As noted, CVC screening orders involved additional challenges because of the complex order modalities and timelines; as a result, these orders were generally not pended via CGS by non-clinician staff. In addition, providers did not always place orders through the CGS but rather elsewhere in the chart because of the process needed to confirm the appropriate CVC-related order.

Some participants noted that certain care gaps did not always appear as expected because of how prior screening results were entered, which could create mistrust. For example, prior lab results from an external provider were not systematically documented in a way that communicated with the CGS, necessitating searching for historic lab results and impacting trust in the tool. A nurse practitioner commented,

*One of the problems that we run into … is that people will scan the colonoscopy report into the GI referral. So that's one of the big problems that we have, so I end up having to print them out. And I can only catch it if I'm asking that patient, “Did you have your colonoscopy done?” Because if I don't ask them, I'm not going to know to look there because I might not always look at their GI reports.* [CHC 3]

## Discussion

Suboptimal cancer screening order rates in CHCs underscore the need to identify effective tools for enhancing CHCs' ability to systematically provide indicated screening orders. This study considered an EHR tool designed to reduce care gaps by streamlining how they are identified and how related orders are placed, by assessing use rates of the tool, the extent to which its use was associated with improved screening order rates, and clinic staff thoughts about its utility. Rates of CRC screening orders were significantly higher among clinics and providers that used the CGS compared to non-users, with no association between CGS use and CVC screening orders. These findings align with other studies showing that EHR tools such as clinician alerts and reminders, screening and result follow-up decision support, and patient outreach can improve cancer preventive care, although the adoption of these tools is highly variable. 7 16 26 27 28 29

The difference in results relating to CRC versus CVC screening orders may reflect the different steps involved in completing those orders. CRC orders are inputted at a given encounter, but completed at a secondary encounter or step. Therefore all that is required at the encounter is to enter the order in the EHR through the CGS. However, CVC screening (Pap test) is often completed when the order is placed and takes time and multiple care steps. If there is not enough time to conduct the Pap at that visit, clinic staff may choose to not enter the order. Another reason for the different findings related to CRC versus CVC screening is that if clinicians opt to refer patients to an obstetrics/gynecologist provider rather than place a CVC screening order, the CGS, which supports screening order provision, not referrals, will be less useful. Additionally, the CVC guidelines' complexity means that related orders must be made by clinicians who first need to verify patient eligibility for a given CVC screening. Research is needed on how CGS-like tools can support CVC screening order processes.

Furthermore, while the differences seen in CRC screening orders are statistically significant, they are not large in absolute numbers, with indicated orders issued at approximately 6% more encounters among the highest CGS users. This is a large relative improvement over the baseline rate of about 10%, but still a suboptimal rate of order provision. Thus, the CGS tool improves CRC screening but the magnitude of the effect in the considered timeframe was small and unlikely to have a meaningful clinical impact. Additional strategies are needed to increase the use of the CGS tool and close gaps in CRC screening. Further research should explore how to optimize CGS-like tools' ability to improve screening order rates, and what other contextual elements support this, such as workflow optimization.

Additionally, while results show that CGS use can improve cancer screening order rates, overall tool adoption was low. Prior studies found similar rates of clinical decision support tool adoption. 16 28 Those who used the CGS found it helpful and easy to use. Barriers to its use included: knowledge gaps, for example, some users did not know how to customize the CGS to fit clinic needs; mistrust in the accuracy of identified care gaps; and potential users were unaware that the tool existed. Similar barriers have been identified in past studies; leadership support, ease of use, staff capacity, and workflow have been commonly reported as barriers/facilitators of such tools' adoption. 16 19 28 29 30

Such barriers might be addressed through user training, which is supported by the finding that clinics that used it provided such training. Potential users might adopt the tool more by training clinic staff to provide needed customization and/or addressing potential pitfalls or perceived inaccuracies. Prior research shows that training is an essential element in enhancing clinical decision-support tool adoption in health centers. 31 32 Yet there are substantial barriers to providing such training, including that training takes time from patient care. 31 32 Research is needed on strategies for improving training content and facilitating training provision.

This study has limitations. Clinicians may use EHR interfaces other than the CGS to order cancer screenings; we assessed only whether rates were associated with CGS use. Because the tool targeted point-of-care order provision, these analyses did not consider whether provided orders were completed, which would not be impacted by the CGS. These factors mean the results are generalizable only to screening order provision that involves an order set. Further, we assessed differences in order rates associated with encounters where the CGS was used for any purpose, rather than for cancer orders specifically. The CGS is designed to present all “care gaps” in one place. Assessing whether users engaging with it for any reason led to higher cancer screening order rates may have biased the results conservatively, as some users likely engaged with the CGS for purposes besides cancer screening orders. Survey respondents' specific clinic roles were not determined, limiting knowledge of how perceptions of the CGS varied by role, and though attendees at the meeting where the survey was conducted were generally representative of the diverse OCHIN network, the survey was opportunistic in nature; its 34% response rate and inability to evaluate which people answered specific questions may have yielded a biased sample. The qualitative data are pragmatically limited to a small sample of tool users whose responses may not be representative of other CHC members regardless of CGS use level. These analyses are descriptive and as in all such analyses may be impacted by residual confounding. These analyses span the first years of the COVID-19 pandemic, in which primary care delivery was often disrupted. While the analyses are designed to consider the impact of the pandemic, this was an unusual period for healthcare delivery, so results may not be representative of what can be expected in usual situations. Future research can build on these preliminary findings.

## Conclusion

In conclusion, a point-of-care clinical decision support tool was associated with higher CRC screening order provision rates, with no association with CVC screening. Findings suggest that maximizing clinical decision support tool use requires leadership buy-in to establish the use of the tool as recommended practice, workflows that integrate tool use by non-clinician staff, and effective training to diverse staff members to improve knowledge and facilitate customization of the tool.

## Clinical Relevance Statement

Promoting the use of the CGS and similar tools to improve cancer screening rates will likely require multifaceted strategies including leadership engagement, workflow design, and training.

## Multiple-Choice Questions

1. What barrier(s) impact(s) adopting a clinical decision tool?
  - Lack of trust in data accuracy
  - Lack of understanding of how to use it efficiently
  - Not knowing it exists
  - All of the above
  **Correct Answer** : The correct answer is option d. All barriers listed above impact the adoption of the CGS tool.
2. What was associated with CGS tool adoption?
  - Higher rates of screening for all cancer outcomes
  - Higher rate of colorectal cancer screening orders
  - Higher rate of cervical cancer screening orders
  - None of the above
  **Correct Answer** : The correct answer is option b. Clinics that had higher rates of CGS tool use also had higher screening orders for colorectal cancer screening.
