# Supplementary material for: The Effect of an EHR Order Set on Cancer Screening Order Rates in Community-Based Health Centers
Source: Appl Clin Inform. 2025 Jun 4;16(3):496–506. doi: 10.1055/a-2524-5076 (PMC12137202; doi:10.1055/a-2524-5076)
Supplement: Supplementary file 1 — Supplementary Material [file 10-1055-a-2524-5076-s202408ra0271.pdf]

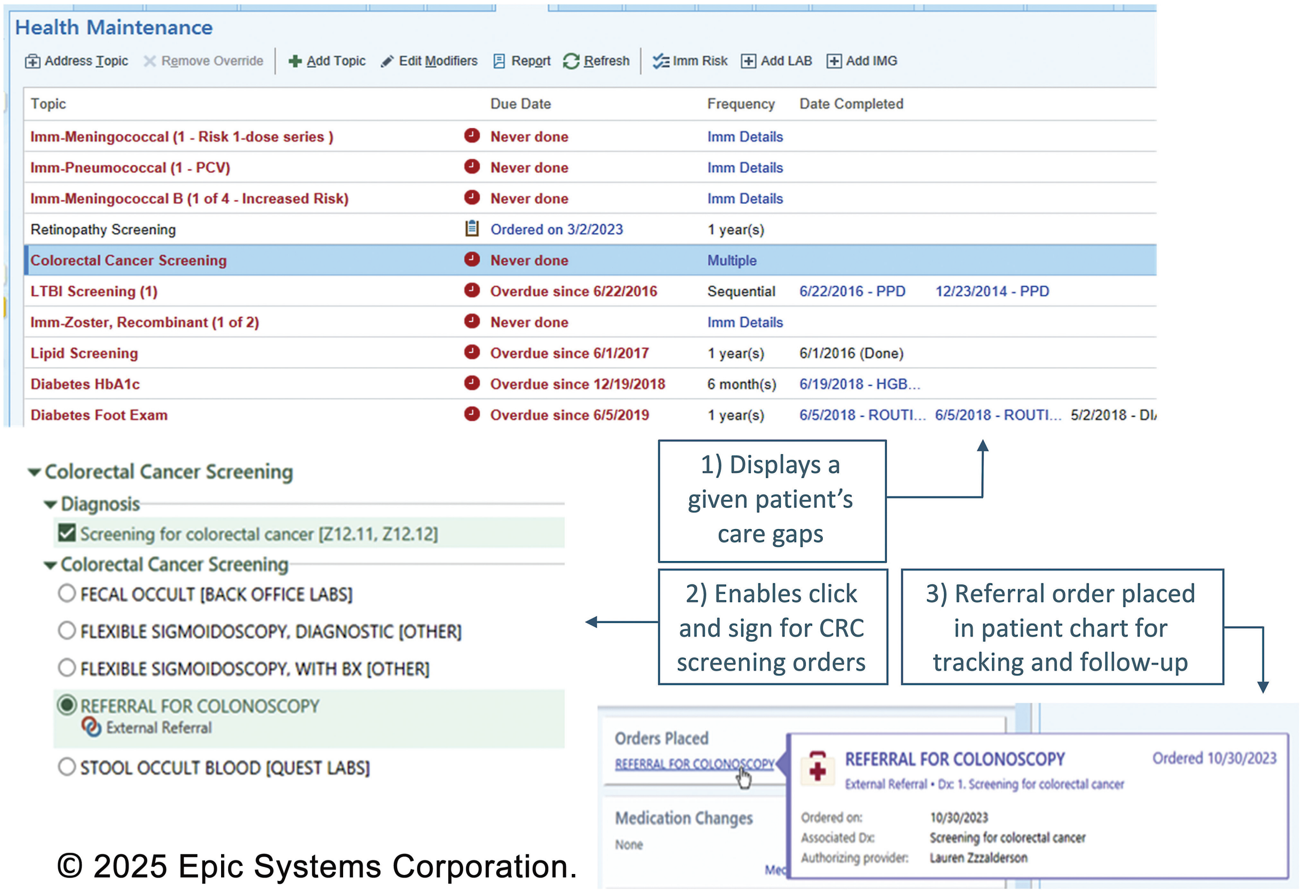

Supplementary Fig. S1 Care gap smartset screenshots (data presented in the figure are imaginary).

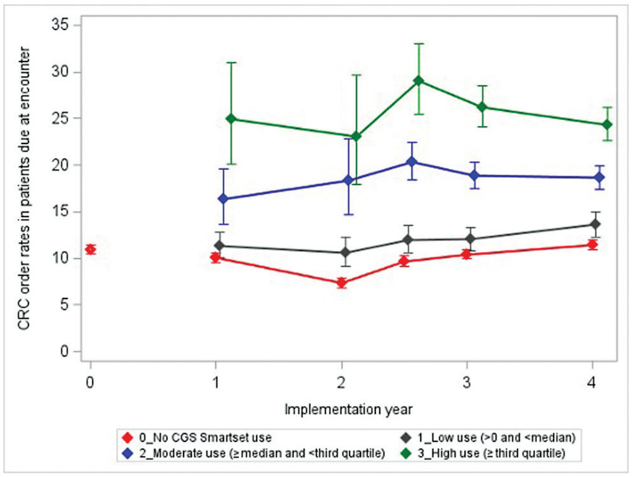

Supplementary Fig. S2 Provider-level CGS use and colorectal cancer screening order rates, 2018–2023. CGS, CGS, care gaps smartest.

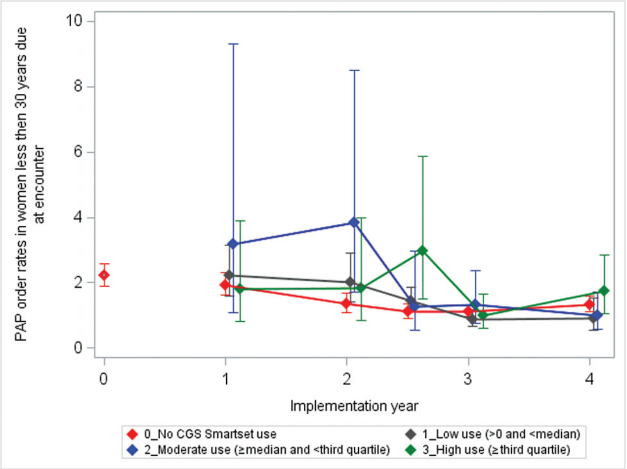

Supplementary Fig. S3 Provider-level CGS use and cervical cancer order rates for women (age <30 years), 2018–2023. CGS, care gaps smartest.

 © 2025. The Author(s).

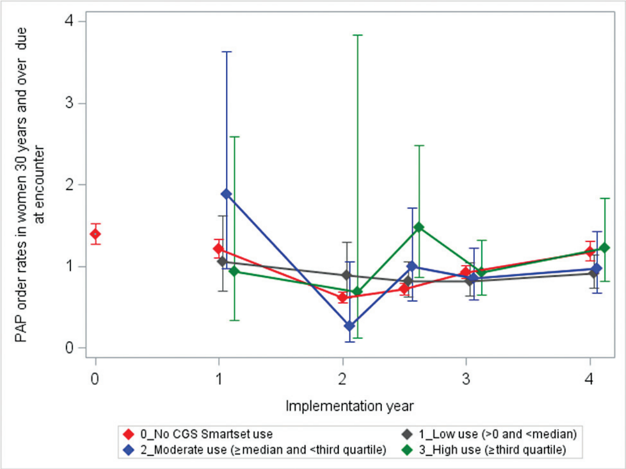

Supplementary Fig. S4 Provider-level CGS use and cervical cancer order rates for women (age ≥30 years), 2018–2023. CGS, care gaps smartest.

 © 2025. The Author(s).
